# The cost of changing physical activity behaviour: evidence from a "physical activity pathway" in the primary care setting

**DOI:** 10.1186/1471-2458-11-370

**Published:** 2011-05-23

**Authors:** Christian EH Boehler, Karen E Milton, Fiona C Bull, Julia A Fox-Rushby

**Affiliations:** 1 Health Economics Research Group (HERG), Brunel University, Uxbridge, Middlesex, UB8 3FG, UK; 2 British Heart Foundation National Centre for Physical Activity and Health, School of Sport, Exercise and Health Sciences, Loughborough University, Loughborough, Epinal Way, Leicestershire, LE11 3TU, UK; 3 School of Population Health, University of Western Australia, 35 Stirling Highway, Crawley WA 6009, Australia

## Abstract

**Background:**

The 'Physical Activity Care Pathway' (a Pilot for the 'Let's Get Moving' policy) is a systematic approach to integrating physical activity promotion into the primary care setting. It combines several methods reported to support behavioural change, including brief interventions, motivational interviewing, goal setting, providing written resources, and follow-up support. This paper compares costs falling on the UK National Health Service (NHS) of implementing the care pathway using two different recruitment strategies and provides initial insights into the cost of changing physical activity behaviour.

**Methods:**

A combination of a time driven variant of activity based costing, audit data through EMIS and a survey of practice managers provided patient-level cost data for 411 screened individuals. Self reported physical activity data of 70 people completing the care pathway at three month was compared with baseline using a regression based 'difference in differences' approach. Deterministic and probabilistic sensitivity analyses in combination with hypothesis testing were used to judge how robust findings are to key assumptions and to assess the uncertainty around estimates of the cost of changing physical activity behaviour.

**Results:**

It cost £53 (SD 7.8) per patient completing the PACP in opportunistic centres and £191 (SD 39) at disease register sites. The completer rate was higher in disease register centres (27.3% vs. 16.2%) and the difference in differences in time spent on physical activity was 81.32 (SE 17.16) minutes/week in patients completing the PACP; so that the incremental cost of converting one sedentary adult to an 'active state' of 150 minutes of moderate intensity physical activity per week amounts to £ 886.50 in disease register practices, compared to opportunistic screening.

**Conclusions:**

Disease register screening is more costly than opportunistic patient recruitment. However, additional costs come with a higher completion rate and better outcomes in terms of behavioural change in patients completing the care pathway. Further research is needed to rigorously evaluate intervention efficiency and to assess the link between behavioural change and changes in quality adjusted life years (QALYs).

## Background

Physical activity is an important contributor to physical and mental well-being, and is also recognised as one of the most important behaviours associated with the prevention of chronic diseases, including coronary heart disease, diabetes, cancer and stroke [1,2]. Despite these positive benefits, data from the Health Survey for England (2008) show that almost 60% of men and approximately 70% of women are insufficiently active to benefit their health [3]. The societal cost of physical inactivity in England alone is estimated to be about £8.2 billion annually [1]. This includes direct costs of treatment for the major lifestyle-related diseases and indirect costs caused through work absenteeism, but excludes the contribution of physical inactivity to obesity, which causes in itself an estimated cost of £2.5 billion annually [1,4]. It is therefore not surprising that interventions to increase physical activity have high priority in public health policy [2].

In 2006, the National Institute for Health and Clinical Excellence (NICE) published public health intervention guidance on physical activity. In this guidance, NICE *"fully endorses the importance of physical activity as a means of promoting good health and preventing disease, and the consequent need to develop comprehensive, multi-sectoral strategies to promote physical activity as part of daily life" *[2]. Existing studies also indicate that various interventions to promote physical activity are cost-effective [5-8]. The interventions assessed include training and support for health practitioners in advising patients to increase physical activity [9], printed resources for patients [10], exercise advice and the opportunity for patients to join exercise groups [11] and supervised exercise training schemes [12]. NICE also identified brief interventions (BI's) as highly cost-effective [2]. These can vary from basic advice, to offering extended and individually tailored consultations to identify and motivate change in physical activity behaviour [2].

Following the publication of NICE Public Health Guidance [2], the Department of Health (DH) in partnership with Natural England and the National Health Service (NHS) London piloted a physical activity care pathway (PACP) [13]. The PACP combines several methods reported to support behavioural change, including BI's, motivational interviewing, goal setting, providing written resources, and follow-up support [2,14-17]. The intervention was implemented in two waves to allow for a rolling start and for lessons learnt from Wave One to inform and improve delivery and implementation in a convenience sample of six London-based general practice surgeries in Wave Two. This paper focuses on Wave Two as experiences from Wave One led to significant modifications of the care pathway. To reflect differences in socio-economic status and patient demographics, practices were selected from a range of localities within the London area [18]. Following the pilot study, the care pathway has since been introduced as 'Let's Get Moving', a policy to promote physical activity commissioned at a local level by Primary Care Trusts (PCT's) within the NHS-system [19]. Although the Department of Health's dissemination plans for the PACP are primarily focused on England, data from this pilot is likely to be of interest to other areas of the UK which have a very similar primary care structure, or indeed other countries using the primary care setting as a catalyst to address physical inactivity prevalence.

Although some international evidence on the cost-effectiveness of elements delivered within the PACP exist [5-8], there is little evidence on the cost or cost-effectiveness of combining these approaches within one intervention package designed for practice implementation in primary care. Stevens et al. (1998) provided initial evidence that a primary care based physical activity intervention would be cost-effective and stressed that the process of patient recruitment was the most important variable cost of delivery [20]. This paper compares the cost of implementing the PACP according to two different recruitment strategies as well as stage of intervention and provides a first indication of how cost varies by patient and centre. In addition, we provide initial insights into the cost of changing patients' physical activity patterns using individual data collected in both recruitment arms at baseline and at three month follow-up. In combination with deterministic and probabilistic sensitivity analyses we consider how these data might inform the efficient delivery of future health promotion of physical activity. The National Research Ethics Service (NRES) advised that this pilot, including its evaluation, falls within the category of 'audit' and did not require local ethics committee (LEC) approval.

The paper first provides a brief description of the PACP intervention protocol (a more detailed description of the overall study design and intervention protocol can be found elsewhere [18]), followed by the methods used for measuring resource use, cost, and behavioural change associated with the PACP. The total programme cost and cost per patient completing the PACP are reported, followed by results on the cost of changing physical activity within each recruitment arm. The extent and causes of differences in costs are considered and a sensitivity analysis presents the impact of alternative assumptions on costs per person completing the PACP and the uncertainty around our estimates of cost-effectiveness. The discussion considers the robustness and generalisability of the findings.

### The PACP intervention

**Figure 1:**
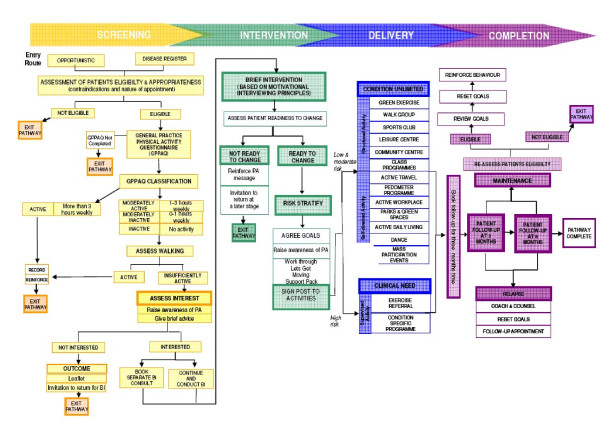
**PACP intervention protocol**.

Figure 1 illustrates the intervention protocol of the PACP. A total of ten health professionals (GP's, nurses and health care assistants) were trained to deliver the PACP and recruited patients over a 12 week period (from January to March 2008, with follow up starting in April). Practices were assigned either to opportunistic recruitment (n = 3), which required health professionals to consider the eligibility of every patient for the intervention during routine practice, or to disease register recruitment (n = 3), which involved contacting patients on the hypertension disease register, via a letter, phone call or text message, to invite them to take part in the PACP.

Inclusion criteria for the PACP specified that; people were aged 16 to 74 years; displayed no contra-indications to exercise, and; it was appropriate to discuss physical activity in the context of the consultation. For those meeting the criteria, practitioners used the 'General Practice Physical Activity Questionnaire' (GPPAQ) to assess physical activity levels [21]. Patients identified as 'insufficiently active', i.e. failing to meet the current UK physical activity recommendation [1,22], were invited to receive a BI, which was delivered by the trained professional either as an extension of the screening consultation (time permitting) or booked as a separate appointment. Patients not interested in a BI were provided with the British Heart Foundation's 'Let's Get Physical' leaflet.

The BI aimed to support patients to change their behaviour by giving advice, setting activity goals, and 'signposting' patients to local physical activity opportunities. The signposting options included local authority leisure services, private clubs, sports and dance, pedometer schemes, outdoor activities and exercise referral schemes. Exercise referral schemes involve directing patients to a service offering an assessment of need, development of a tailored physical activity programme, monitoring of progress and follow up support [2]. Patients were also provided with the 'Let's Get Moving' resource booklet which contained information on the benefits of being physically active as well as opportunities for physical activity locally. Finally, health professionals agreed individual activity goals with the patient. The PACP protocols specified a patient follow-up consultation at three months, as an opportunity to assess and reinforce the patients' change in lifestyle and to review the patients' activity goals.

## Methods

### Framework

The aim of the analyses is to describe how costs falling on the NHS vary by stage of implementation of the intervention and method of delivery; and to estimate the cost of changing physical activity behaviour within each recruitment arm. Costs falling on other organisations (e.g. the British Heart Foundation booklet) or people (i.e. time or money costs falling on patients) were excluded, and no costs associated with the actual physical activity adopted were accounted for. Costs are focussed solely on promotion of physical activity within a GP surgery and for the year 2007. The NHS perspective was chosen because we focus on two competing strategies within the same primary care setting [23], and because a broader 'societal perspective' would have had to rely on weak data and strong assumptions. Our choice of perspective is in accord with the 'reference case' of the English 'NICE guideline for technology appraisal' and several other health technology assessment guidelines around the world [24,25].

To allow a more accurate allocation of overhead costs and understanding of causes of variation in costs [23], a time driven variant of activity based costing (ABC) was used to facilitate the measurement of resources consumed by individual patients and to allocate single cost items to each patient [26,27]. Time driven ABC requires, in principle, two parameters: the time required to perform an activity and the unit cost of resources [27]. This was operationalised by:

1. The individual steps of each patient through the pathway protocol (e.g. patient entry route, attending a screening consultation, receiving the brief intervention, receiving a follow-up consultation);

2. The actual resources used per patient at each stage of the pathway (e.g. time spent in screening consultation, brief intervention and follow-up consultations; and time spent on support activities including contacting patients);

3. The unit cost of the resources supplied within the pathway (e.g. unit cost of GP's and associated members of staff, unit cost of 'Let's Get Moving' booklet).

Data on physical activity behaviour was used from 70 patients completing the PACP, of which full data for both baseline and three month follow-up was available in 46 patients. Two questions were asked at both occasions:

1. 'In the past week, on how many days have you accumulated at least 30 minutes of moderate intensity physical activity such as brisk walking, cycling, sport, exercise, and active recreation. Do not include physical activity that may be part of your job or usual role activities.'

2. 'How much time in total do you estimate you spent participating in moderate intensity physical activity last week?'

To account for potential selection bias resulting in time-invariant differences between both treatment groups, behavioural change was estimated using a regression based 'difference in differences' using self reported time spent on physical activity in the week prior to the baseline assessment and the week prior to the three month follow-up [28].

### Data collection

Individual patient data was collected directly by the trained practitioners using specifically designed templates delivered through the Egton Medical Information System (EMIS) or similar software systems (for more information see [18]). Templates were designed to reflect the consultation steps involved in delivering the care pathway: screening; brief intervention; and three-month follow-up. Data were downloaded from EMIS using a MIQUEST search which was either conducted 'locally' at the practice or 'remotely' via the PCT. The cost analysis used data on completion of activities, delivery of booklets, and time spent per consultation.

To obtain data on the use of resources at each stage of the PACP, a survey of practice managers was completed with the help of other administrative and health care professionals. Staff were asked about support activities, for example, who was responsible for contacting patients as well as the time taken (survey form available in final report [29]). The survey was followed up by telephone to either gain missing information or to deal with queries. This approach facilitated high compliance and ensured that no answers were missing or misunderstood. Table 1 shows the level of detail to which 'activity' was defined as well as the nature and source of data collected for this costing exercise.

**Table 1:** Data points utilised in the costing model

Variable	Type	Level	Categories	Data Source
Patient Recruitment method	Binary	Patient	Disease register screening/Opportunistic screening	EMIS

Initial patient contact	Categorical	Patient	Opportunistic/letter/letter attached to existing recall letter/phone/text message	EMIS

GPPAQ assessment completed?	Binary	Patient	Yes/No	EMIS

Patient activity index?	Binary	Patient	Active/inactive	EMIS

Patient interested in BI?	Binary	Patient	Yes/No	EMIS

Patient ready to change?	Binary	Patient	Yes/No	EMIS

BI consultation booked/continued?	Categorical	Patient	Booked/continued/patient not interested	EMIS

BI consultation attended?	Binary	Patient	Yes/No	EMIS

Patient received BHF leaflet?	Binary	Patient	Yes/No	EMIS

Patient received support package?	Binary	Patient	Yes/No	EMIS

Patient contact for follow-up?	Categorical	Patient	Letter/Letter attached to existing recall letter/phone/text message	EMIS

Follow-up consultation attended?	Binary	Patient	Yes/No	EMIS

Health professional conducting: 1) Screening consultation 2) Brief Intervention 3) Follow-up	Categorical	Patient	General Practitioner/Nurse (NHS pay band 5)/Nurse (NHS pay band 7)/Healthcare assistant (NHS pay band 2)/Healthcare assistant (NHS pay band 3)	EMIS

Time taken for: 1) Screening consultation 2) Brief Intervention 3) Follow-up	Continuous	Patient		EMIS

Member of staff responsible for 1) screening disease registers 2) contacting patients (via letter, phone, text message) 3) booking appointments	Categorical	Centre	Receptionist (NHS pay band 2)/Medical Secretary (NHS pay band 3)/Medical Secretary (NHS pay band 4)/Practice Manager (NHS pay band 5)/Practice Manager (NHS pay band 6)	Survey

Time required for 1) screening disease registers 2) contacting patients 3) booking appointments	Continuous	Centre		Survey

To increase the generalisability of results, all estimates of unit costs represent national averages rather than London weighted unit costs. Unit cost estimates for GPs and practice nurses (including their share of overheads and capital costs) were taken from Curtis (2007) [30]. As estimates of unit cost for healthcare assistants, receptionists, medical secretaries and practice managers were not available in Curtis (2007), unit costs for these categories of staff were derived using the same methods, sources and assumptions given in Curtis (2007). Table 2 shows details of both unit staff costs and other unit costs for non-staff inputs, included promotional materials and costs of contacting patients. The 'Let's Get Moving' support booklet was produced on a low scale for the pilot project and consequently has a high cost per unit (£12.91/pack) compared with the cost quoted for a national roll out (£0.32/pack).

**Table 2:** Unit cost estimates

Resource supplied	Resource use measure	Unit cost (2007)	Source for unit cost
**Health practitioners and other staff at participating centres **(estimates reflect salaries, salary on-costs, qualifications*, practice overheads and capital costs)			

GP*	time spent per patient	£100.79/h	Curtis (2007)

Nurse*	time spent per patient	£26.41/h	Curtis (2007)

Nurse (intermediate level)*	time spent per patient	£30.89/h	Curtis (2007)

Nurse (advanced)*	time spent per patient	£37.84/h	Curtis (2007)

Healthcare assistant	time spent per patient	£15.31/h	own calculation based on Curtis (2007)

Healthcare assistant (higher level)	time spent per patient	£16.79/h	own calculation based on Curtis (2007)

Receptionist	time spent per support activity	£15.31/h	own calculation based on Curtis (2007)

Medical secretary	time spent per support activity	£16.79/h	own calculation based on Curtis (2007)

Medical secretary (higher level)	time spent per support activity	£19.15/h	own calculation based on Curtis (2007)

Practice manager	time spent per support activity	£22.15/h	own calculation based on Curtis (2007)

Practice manager (higher level)	time spent per support activity	£26.42/h	own calculation based on Curtis (2007)

**'Let's Get Moving' support booklet**			

LGM-pilot: 6 pp with pocket + 8 pp stitched text, 350 gsm/130 gsm coated silk	direct cost, assigned to each patient receiving the support pack	£12.91/pack	Department of Health

National roll out: Amend artwork to create a booklet of 12 pp text + 4 pp cover, no pocket (500,000 packs), 250 gsm/130 gsm coated silk	direct cost, assigned to each patient receiving the support pack	£0.32/pack	Department of Health

**Other cost items**			

Stamp 1^st ^class	Assigned to each patient contacted by mail	£0.36/stamp	Royal mail price finder

Stamp 2^nd ^class	Assigned to each patient contacted by mail	£0.27/stamp	Royal mail price finder

Charge per text message	Assigned do patients contacted by text message	Free of charge	Survey

Phone charge per minute	Assigned to patients contacted by phone	£0.03-£0.09/min.	Survey

Cost/hour of member of evaluation team who delivered ongoing practice support	Time spent with practice support per GP surgery	£47.00/h	Full Economic Costing

Cost of practitioner training	Equally allocated to each participating centre	£710/centre	Contract with Health Consultant delivering practice training

In order to deliver the PACP, additional training and support was given to the health practitioners involved. The training took place over two days as a one-off session, delivered by a consultant with a clinical background in physical activity and smoking cessation and a research background in physical activity and behaviour change. The cost of this was divided equally between participating centres (£710/centre, cost provided by the Department of Health). Further support was given by telephone to help with any difficulties arising during the first weeks of delivery of the PACP at a rate of £47/hour for a maximum of two hours. These costs are treated as overheads.

Data availability through EMIS for four centres was excellent and any missing data values were resolved via telephone. However, one centre did not provide any data on BI's, and another centre failed to provide any follow-up data. As these problems were not resolvable through following-up centres or using data imputation techniques, we excluded both centres from the analyses. The analysed sample therefore consists of two disease register centres and two centres following an opportunistic screening process.

### Methods of analysis

EMIS data, survey data and cost data were combined using templates in Microsoft Excel. The resulting dataset contained cost estimates of each individual patient processing through the PACP intervention, which was then used to estimate total cost and mean cost per participating centre and delivery model. The cost of practice training and set-up advice were only considered to estimate the total cost of the PACP pilot. As this cost component was dominant only due to the short study implementation period and as its relevance would diminish on a marginal level with increasing patient throughput, cost of practice training and set-up advice was not considered in further analyses of patient level costs. Descriptive statistics show the distribution of cost per patient completing the PACP for both delivery models and each stage of the intervention process. Two-sample t-tests, adjusting for clustering, tested whether consultation time and cost per patient differed significantly between opportunistic and disease registry sites.

The regression based 'difference in differences' applied an ordinary least squares (OLS) model of the form [28]:(1)

The dependent variable (*Yi*) is the self reported time in physical activity of each patient, 'T' is a time dummy variable coded 1 for 'follow-up' and 0 otherwise, 'DR' is a treatment dummy coded 1 for 'disease register screening' and 0 otherwise, and 'T*DR' is a time-treatment interaction term. The coefficient *β*_0 _estimates the time spent on physical activity in opportunistic centres at baseline, *β*_1 _estimates the change in physical activity patterns in opportunistic centres over time, β_2_ captures the difference between recruitment arms at baseline, and β_3_ estimates the difference in differences in time spent on physical activity between both recruitment arms at baseline and three month follow-up. Analyses were undertaken using STATA 10.

Data on cost, completion rate (defined as the percentage of patients recruited who completed the care pathway in both recruitment arms) and behavioural change was combined to estimate the cost to increase patients' time spent on moderate intensity activity to 150 minutes per week, which accords the current, Government endorsed, physical activity recommendation [1] and the recommendation of the 'British Association of Sports and Exercise Sciences [31]. Incremental cost effectiveness was calculated as:(2)

In equation 2, *C*_*DR *_and *C*_*OPP *_are the mean cost per patient completing the PACP in each recruitment arm, which already accounts for the cost of patients not completing the care pathway. Δ*T*_*DR *_and Δ*T*_*OPP *_represent the change in self reported activity levels in each group, whilst *R*_*DR *_and *R*_*OPP *_denotes the completion rate in each delivery arm.

The impact of factors suspected to significantly influence cost estimates across delivery models were analysed further through sensitivity and scenario analyses. The factors considered were: 1) assuming a national roll out of the PACP with pilot level costs except with the cost of the 'Let's Get Moving' resource booklet reduced from £12.91 to £0.32 per pack; 2) an assumption that all patient consultations could be delivered by healthcare assistants as opposed to GP's or nurses; 3) an assumption that all support activities could be delivered by receptionists (NHS pay band two); 4) the simultaneous change of all the above factors; and 5) in addition to the assumptions of scenario 4 - assuming an equal time to deliver patient consultations within each delivery model. Two-sample t-tests, adjusting for clustered data, were repeated after each analysis to test whether differences in mean costs across delivery models were significant. To demonstrate uncertainty in estimates of cost of changing patients' physical activity behaviour, we performed probabilistic sensitivity analysis. Gamma distributions were assumed for both cost and physical activity data using methods of moments and data was generated for a hypothetical cohort of 1000 patients in each recruitment arm [32]. As there were no data on behavioural change in patients who failed to complete the care pathway protocols, we conservatively assumed zero impact of the PACP on physical activity levels in these individuals.

## Results

The total cost for delivering the PACP in the four centres was £18,231, which covered 411 screened patients of whom 75 provided follow-up data at 12 weeks. Practice training and set-up advice was the largest cost contributor (£11,349). From the total cost of delivery, £8,852 (49%) occurred at opportunistic sites and £9,379 (51%) at disease register practices. While opportunistic sites recruited far more patients to enter the PACP, patient compliance was higher throughout the course of the intervention at disease register centres (see Table 3).

**Table 3:** Mean time and mean cost per patient within delivery models (£ sterling, 2007)

	Delivery Model	Screening	Brief Intervention	Follow-up	Total across intervention
**Number of patients**	**OPP**	334	181	54	54

**Number of patients**	**DR**	77	68	21	21

**Completion rate**	**OPP**	100%	54.2%	16.2%	16.2%

**Completion rate**	**DR**	100%	88.3%	27.3%	27.3%

**Mean time/patient (min)**	**OPP (SD)**	2.21 (1.15)	3.63 (0.95)	2.37 (0.85)	28.23 (1.92)

**Mean time/patient (min)**	**DR (SD)**	3.14 (4.95)	17.81 (7.01)	8.38 (5.84)	77.57 (13.77)

**Mean difference (p)**		0.93 (0.78)	14.18* (0.0665)	6.01 (0.1467)	49.34** (0.04)

**Mean cost/patient (£)**	**OPP (SD)**	1.51 (1.49)	11.71 (5.77)	4.35 (1.73)	53.22 (7.82)

**Mean cost/patient (£)**	**DR (SD)**	17.43 (9.07)	35.08 (18.99)	13.32 (10.37)	190.84 (38.98)

**Mean difference (p)**		15.91** (0.0482)	23.37 (0.2521)	8.98 (0.328)	137.62** (0.047)

* significant at 10% level; ** significant at 5% level; *** significant at 1% level

The mean consultation time was significantly higher at disease register practices compared with centres using opportunistic screening (77.6 min (SD 13.8) vs. 28.2 min (SD: 1.9)). This pattern held for each part of the pathway, although the only difference in mean times that reached statistical significance was for the delivery of the brief intervention. The cost per patient completing the PACP was also significantly higher at disease register practices (£190.80 (SD 39) vs. £53.20 (SD 7.8)). This pattern also held across each part of the pathway, although it only reached statistical significance for the screening consultation.

**Figure 2:**
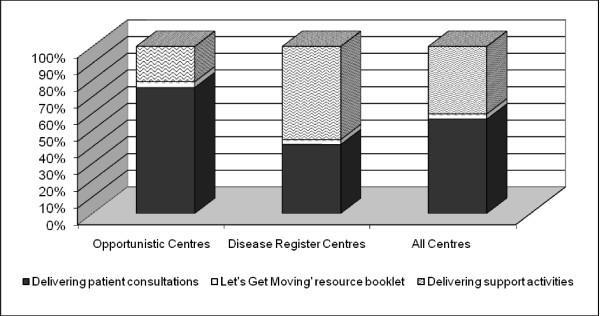
**Cost of activities within the PACP as percentage of total cost (less training and set up advice)**.

The cost of activities as a percentage of total cost (less training and set-up advice) is shown by delivery model in Figure 2. The balance of percentages differed by delivery model, with patient consultations having the largest share in opportunistic sites and support services (i.e. screening disease registers, contacting patients, booking appointments) the largest share in practices recruiting from disease registers. The 'Let's Get Moving' resource booklet was responsible for 40.6% of total cost at opportunistic sites and 23.9% at disease register centres.

Self reported behavioural change in opportunistic centres between baseline and three month follow-up amounts to 9.8 (SE: 8.2) minutes per week, whilst patients in disease register centres reported an increase in physical activity levels by 91.1 (SE: 15.1) minutes in the week prior to the follow-up appointment. The difference in differences in physical activity levels between both recruitment arms is 81.3 (SE: 17.2) minutes of self reported moderate intensity physical activity. Combining these estimates with the observed completion rate in each recruitment arm and the estimated cost per patient completing the PACP (both obtainable from table 3, column 6) leads to an incremental cost of £886.50 to increase self reported physical activity levels to 150 minutes of moderate intensity activity per week when comparing disease register screening with opportunistic patient recruitment.

The sensitivity analysis on cost showed that the impact of changing one factor had varying impacts on the cost per patient by method of delivery. Table 4 shows that using the national 'roll out' cost for the LGM booklet has the largest impact on reducing costs for opportunistic screening and that ensuring patient consultations are delivered by health care assistants leads to the greatest cost reduction for disease register sites. Asking receptionists to deliver all support services has least impact on cost reduction in either mode of recruitment.

**Table 4:** Sensitivity and scenario analysis

		Opportunistic		Disease Register		
**Scenarios**		**Mean cost/patient compl. PACP (SD)**	**% change compared to base case**	**Mean cost/patient compl. PACP (SD)**	**% change compared to base case**	**Differences in mean cost across delivery models (p)**^**+**^

**0**	Base case (Figure 3a)	£53.22 (7.82)	--	£190.84 (38.98)	--	£137.62** (0.047)

**1**	National rollout of 'Let's get Moving' resource booklet	£20.58 (3.52)	-61.33%	£155.47 (35.18)	-18.53%	£134.89** (0.0401)

**2**	All consultations delivered by healthcare assistant	£44.94 (6.50)	-15.56%	£128.29 (23.74)	-32.78%	£83.35** (0.0451)

**3**	All support activities delivered by receptionist (NHS pay band 2)	£52.68 (7.81)	-1.01%	£178.29 (35.50)	-6.58%	£125.61** (0.0466)

**4**	Altering variability factors 1 to 3 simultaneously (Figure 3b)	£11.76 (1.20)	-77.9%	£80.36 (16.78)	-57.89%	£68.60** (0.0305)

**5**	Changing all factors simultaneously across centres plus controlling for differences in mean consultation time across practices (Figure 3c)	£11.76 (1.20)	-77.9%	£35.64 (4.96)	-81.32%	£23.88*** (0.0089)

* significant on 10% level; ** significant on 5% level; *** significant on 1% level

**Figure 3:**
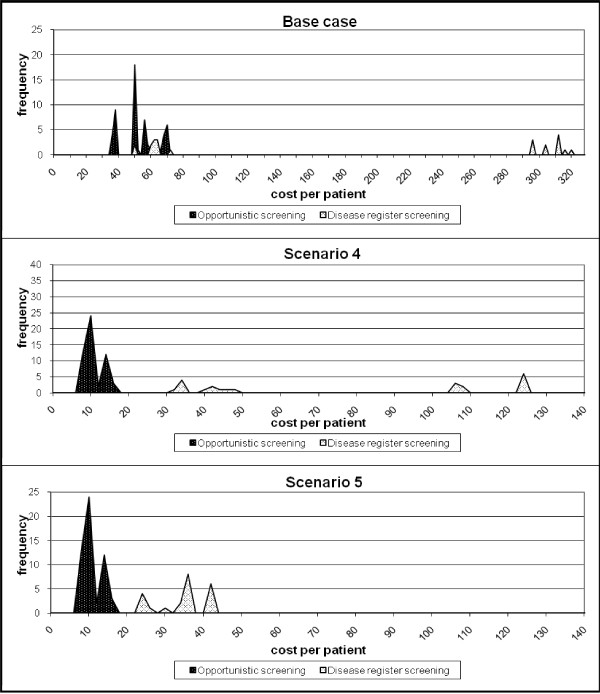
**Distribution of cost per patient completing PACP**.

The scenario analyses show that altering all three individual cost reductions would lead to a 78% cost saving at opportunistic sites and a 58% cost saving at disease register sites. Figure 3 shows the similarity in distribution of costs in centres operating opportunistic screening and the divergence between disease register screening sites, and hence the importance of accounting for cluster effects. Table 4 shows that the cost savings never change the conclusion that opportunistic screening is significantly less costly than using disease registers to screen and enter patients into the PACP. This conclusion is robust even when controlling for differences in the mean time of delivering patient consultations (decreasing time by 56% and 68% in the two disease register centres) between participating practices (see Scenario 5, Table 4 and Figure 3c) as the difference in cost per patient remains significantly different at the 1% level.

**Figure 4:**
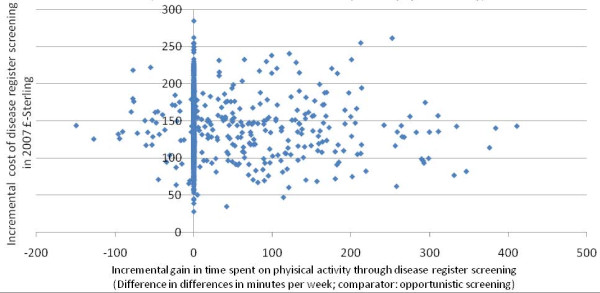
**Probabilistic sensitivity analysis of incremental cost of changing physical activity behaviour in minutes per week**. Figure 4 takes into account that information on behavioural change is missing for anyone who did not present for the follow-up appointment. Conservatively, for these cases we assumed a difference in behavioural change of zero, which explains the huge number of estimates being 'lined up' along the vertical axis of the cost-effectiveness plane.

Probabilistic sensitivity analysis on the incremental cost of changing physical activity patterns shows the extent of uncertainty in cost-effectiveness estimates. As only direct costs of the care pathway were considered and no potential cost-offsets through future disease being avoided by increasing physical activity levels were included, all estimates naturally fall within the north-west and north-east quadrants of the cost effectiveness plane. The large number of estimates being scattered on the vertical axis occur due to the conservative assumption of zero change in physical activity patterns in patients who failed to complete the care pathway protocols. All in all, Figure 4 depicts the great uncertainty surrounding the results.

## Discussion

This research shows that the PACP has the potential to deliver a combination of physical activity interventions already deemed cost-effective at a mean cost per patient completing the care pathway of £53 (SD 7.8) in opportunistic centres and £191 (SD39) at disease register sites. The cost per patient completing the PACP in opportunistic sites remains lower than in disease register sites, even after controlling for the differential unit cost of support staff and healthcare professionals and for differences in the mean time to deliver patient consultations. Moreover, assuming a positive relationship between the experience of the motivational interviewer and the intervention's effectiveness as well as between the time and number of patient consultations and behavioural change (e.g. [16]), scenario 5 could be argued to resemble a cost minimization analysis - in the sense that it controls for observable factors likely to have a systematic effect on the intervention's effectiveness. Doing so led to a large decrease in cost per patient within disease register centres. Nevertheless, opportunistic screening remained less costly with a statistically highly significant difference in mean cost between delivery models. This finding is consistent with NICE's costing report on brief interventions, which states that *"the use of existing appointments and contact with sedentary patients will minimise the cost impact of implementing brief interventions" *[33].

However, our findings also indicate that disease register screening is likely to have better outcomes in terms of completion rate and behavioural change, which leads to an incremental cost-effectiveness estimate of £886.50 to increase self-reported physical activity levels to 150 minutes of moderate intensity activity per week when compared to opportunistic patient recruitment. The important question is why disease register screening appears to be more effective in terms of behavioural change. We believe that there are two possible answers to this question: First, the data may be subject to self-selection bias due to the recruitment strategies applied. Disease register screening potentially recruits patients with a higher intrinsic motivation to change their physical activity behaviour as these patients need to pick up on an invitation to book an appointment with their GP. This higher intrinsic motivation for behavioural change may account for part of the observed differences in both completion rate and behavioural change estimates, which is independent of all the assumptions made in scenario 5. This self-selection effect in disease register sites might even outweigh the effect of health professionals in opportunistic sites who also reported making subjective decisions regarding which patients would be appropriate for the programme and tended to recruit patients who were motivated towards physical activity and therefore likely to take up the opportunity to participate in the PACP. In conclusion, selecting patients who are more motivated towards changing their physical activity behaviour may have a crucial impact on cost-effectiveness. Though, this effect may also raise concerns over the viability of the PACP to address inequalities as evidence suggests that patients who are more likely to take up preventative health services tend to come from more advantaged backgrounds [34].

Secondly, findings might relate to the time spent by practitioners with their patients, a feature which significantly differed across recruitment arms. The shorter time spent delivering the care pathway in 'opportunistic' practices is likely to reflect the time constraints of delivering the care pathway within usual practice. Practices which recruited via the disease registers were able to book, in advance, longer consultations to accommodate the care pathway steps and components [29]. In general, there is evidence of a positive relationship between the effectiveness of brief interventions and the time spent by practitioners with their patients (e.g. [16]). And specific to the PACP, whilst data revealed that all steps were conducted in patient consultations in both recruitment arms, differences in time might reflect '*variations in both the content and quality of LGM delivery*' which is corroborated by the qualitative findings of this study reported elsewhere [18]. This, in turn, may be responsible for some of the increment in effectiveness observed in disease register practices.

As the pilot study design was lacking a comparison group to observe patients who did not undergo the intervention, we are not in the position to calculate the incremental cost-effectiveness of each recruitment strategy versus 'doing nothing'. However, to allow a comparison of our figures with other studies assessing similar public health interventions to increase physical activity, this would be the comparator of choice. For this reason, we assumed no change in cost or time spent on physical activity for a hypothetical 'no intervention' group, and this led to estimates of cost of increasing physical activity in one patient to the recommended target level of 150 minutes of moderate intensity activity per week of £1151.01 in disease register sites and £5038.63 in opportunistic centres. Our estimates are similar to those reported elsewhere after converting international data to 2007 £-Sterling using World Bank specific GDP-deflators and purchasing power parity (PPP) conversion rates [35]. For example, Elley et al. (2004) assessed the cost-effectiveness of verbal advice and a written exercise prescription given by general practitioners, with telephone follow-up from an exercise specialist [36]. They reported programme-cost per patient of NZ$170 (£84.51, 2007 £sterling) from a funders perspective and incremental cost of converting one additional 'sedentary' adult to an 'active' state over a 12-month period of NZ$ 1,756 (£873.00, 2007 £sterling). Sevick et al. (2007) assessed the effectiveness of interventions delivering theory-based, motivationally tailored individualized feedback to sedentary adults, with the goal of increasing physical activity [10]. Similar to our analysis, cost estimates included personnel time for delivering the intervention, curriculum materials, printing, postage and facility costs. The print intervention within this study was estimated to cost US $480 (£332.60, 2007 £sterling) at 12 months, with US $955 (£661.80, 2007 £sterling) spent to successfully engage one participant in a more active lifestyle [10]. Lindgren et al. (2003) estimated the cost-effectiveness of dietary and/or exercise advice both from a health care payers and a societal perspective [11]. Although they concluded that dietary advice dominates exercise advice, the latter was deemed cost-effective compared with no intervention. The PACP combines several of these approaches to promote physical activity, and our results suggest that this combined approach may also be cost-effective, although we strongly suggest that further research is needed to provide more robust estimates of intervention effectiveness and efficiency, and to assess the link between behavioural change and changes in quality adjusted life years (QALYs).

A key question remains at the end of this study: would the assumption that opportunistic screening is less costly, but also less cost-effective than screening by disease register hold when rolling the pathway out in a national context, such as with the planned dissemination of 'Let's Get Moving'? This is particularly important to consider given the weakness of the effectiveness data, the unfortunate exclusion of two participating centres and that the low number of participating sites, characteristic of a pilot, were also relatively large metropolitan surgeries.

In terms of cost per patient completing the PACP, the principal reason for accepting that our conclusion would hold is that the relatively large difference in mean cost of £24 per completing patient remained statistically significant even after a series of stringent scenario analyses (e.g. assuming a much lower price for the resource pack, assuming the same delivery time per patient consultation or that the PACP was delivered by the same health care professional). Basing costs on national rather than London weighted estimates should also improve generalisability and opportunistic screening not only involves much less administrative effort but also significantly less support staff in delivery, so costs might not rise substantially in other locations. External validation of the cost of disease registers is provided in Stevens et al. (1998) [20], whose intervention arm shows some similarities to the disease register arm in our study; for which we reported costs of £190.84 per patient. Increasing comparability with our study through excluding the cost of unused exercise development officer time and updating values to 2007 using World Bank country specific GDP-deflators led to an estimated cost per completer of about £197. Finally, our conclusion is also in accord with Stevens et al (1998) who state that the recruitment process was the most important aspect of the intervention.

In terms of practice profiles, we are aware that the profile of surgeries nationally may not match those of the pilot sample and that, in particular, smaller surgeries staffed largely by GPs may have less scope to substitute care to other health professionals and that this might in turn mean that cost per patient is likely to increase proportionally more in such sites for opportunistic compared with disease register screening. Though, further analysis showed that whilst cost per patient would increase by proportionally more (38% vs.19%) if the service was delivered only by GPs, the increase still did not shift per patient cost for the opportunistic screening to that of the disease register and costs remained significantly different.

In terms of behavioural change and cost-effectiveness we are less confident in the robustness of our findings, which is why we strongly recommend further research to provide more robust estimates of intervention effectiveness and efficiency. There are several reasons for concern: First, estimates of behavioural change in both treatment arms were based on self reported physical activity data. These data are susceptible to different sources of bias which we could not assess any further due to the low numbers of patients completing the PACP in both treatment arms. Secondly, behavioural change was assessed using data from only 70 individuals completing the PACP, whilst full data for both appointments was available in just 46 patients, meaning that our findings are based on very weak effectiveness data; and in the absence of any follow-up data for other patients, we had to assume zero change in physical activity levels in patients who did not complete the PACP (i.e. attend the follow-up appointment). This may have led to an underestimation of treatment effectiveness on the one hand, but it is also possible that further data would increase the variation around point estimates. Thirdly, though our choice of perspective meant that patient cost did not fall within the scope of this exercise, we do regret that we were unable to collect data on this cost item as recent evidence suggests that patient costs may be an independent explanatory of the demand for exercise [37]. In particular, if patients face increased costs by a programme like this or if patient costs differ between groups, this might explain differences in take-up rates, which consequently affects the cost-effectiveness of the intervention [37]. Finally, it can be argued that any behavioural change induced by the intervention may be short term and that a second follow-up appointment later in time would have been required to confirm findings from the three month follow-up. Assuming a decrease in physical activity levels after the intervention period would result in overstated estimates of intervention effectiveness and cost-effectiveness.

## Conclusion

This study collected and utilised individual patient cost data of a package of interventions designed to increase physical activity levels in sedentary patients, including practitioner training, brief interventions, motivational interviewing, goal-setting, written resources and follow-up support in the context of two alternative methods of recruitment. In addition, it provided us insight into the potential cost of changing physical activity behaviour in each recruitment arm. Opportunistic screening was found to deliver the PACP at a lower cost than disease register screening, after controlling for systematic differences between the two study arms and alternative assumptions about national roll out of the programme. However, opportunistic screening is also found to be less effective, though there are significant concerns regarding the robustness of self reported behavioural change data collected within this pilot study. Therefore, further research on linking individual patient costs to outcomes in terms of activity, compliance and quality of life is strongly recommended.

## Competing interests

All authors declare that they have no conflict of interests, including any financial, personal or other relationship with other people or organizations within five years of beginning this study. Funding for this study was from the Policy Research Programme of the Department of Health. The views expressed in the publication are those of the authors and not necessarily those of the Department of Health

## Authors' contributions

CB developed the design for this study and tools for data collection, undertook data analysis, drafted the first manuscript and coordinated its revision. KM helped develop tools for data collection, supported data collection and assisted in drafting and revising the manuscript. FB developed the overall design for the PACP study and assisted in drafting and revising the manuscript. JFR directed the project and assisted in drafting and revising the manuscript. All authors read and approved the final manuscript.

## Pre-publication history

The pre-publication history for this paper can be accessed here:

http://www.biomedcentral.com/1471-2458/11/370/prepub
